# Effect of Rain-Shelter Cultivation of *Vitis vinifera* cv. Cabernet Gernischet on the Phenolic Profile of Berry Skins and the Incidence of Grape Diseases

**DOI:** 10.3390/molecules18010381

**Published:** 2012-12-27

**Authors:** Jiang-Fei Meng, Peng-Fei Ning, Teng-Fei Xu, Zhen-Wen Zhang

**Affiliations:** 1 College of Enology, Northwest A&F University, Yangling 712100, Shaanxi, China; E-Mails: mjfwine@nwsuaf.edu.cn (J.-F.M.); hebeijiangfeim@nwsuaf.edu.cn (P.-F.N.); 2 Institute of Biology II, University of Freiburg, Freiburg 79104, Germany; E-Mail: tengfeixu1035@gmail.com; 3 Shaanxi Engineering Research Center for Viti-Viniculture, Yangling 712100, Shaanxi, China

**Keywords:** Cabernet Gernischet, rain-shelter cultivation, berry skins, phenolic compounds, grape diseases

## Abstract

Rain-shelter cultivation is an effective cultural method to prevent rainfall damage during grape harvest and widely applied in the Chinese rainy regions. In this study we investigated the effect of rain-shelter cultivation on grape diseases and phenolic composition in the skins of *Vitis vinifera* cv. Cabernet Gernischet grape berries through the comparison with open-field cultivation at two vintages (2010 and 2011). The results showed that rain-shelter cultivation reduced the incidence of grape diseases significantly and delayed the maturation of Cabernet Gernischet fruits. With regards to most of the phenolic compounds identified in this study, their content in grape samples under rain-shelter cultivation was decreased compared to those under open-field cultivation. However, rain-shelter cultivation stimulated the accumulation of dihydroquercetin-3-*O*-rhamnoside in grape skins during grape maturation. These were related with micrometeorological alterations in vineyards by using plastic covering under rain-shelter cultivation. It suggests the rain-shelter cultivation makes possible the cultivation of “Cabernet Gernischet” grapes in an organic production system, for providing a decrease in the incidence of diseases and the dependence on chemical pesticides in the grape and wine industry.

## 1. Introduction

*Vitis* is widely cultivated around the world. The grape planting area in China has increased rapidly in recent years, and it is forecast to reach 560 mha in 2011, ranking fourth in the World, according to the OIV statistical report on world vitiviniculture [1]. However, China’s grape and wine industry is now confronted with a tough problem. Located on the Pacific Ocean West bank, most of China enjoys a marked continental monsoonal climate, characterized by hot and rainy summer-autumns, cold and dry winter-springs. This is unfavorable for grape growth, sugar accumulation, organic acid degradation, and phenolic compound formation under such climatic conditions, which seriously hinders the development of the grape and wine industry in China, and improvement of China’s wine quality.

In order to overcome the disadvantages caused by local climate conditions, installation culture techniques, also called protected cultivation, was recently introduced to viticulture in China. Installation culture techniques refers to a fruit culture practice using an artificial microclimate that meets fruit growth requirementz in some installations in the case of an unfavorable natural environment for fruit growth [2]. Rain-shelter cultivation is a simple form of installation cultivation through building shelters to prevent the influence of rain on the crop. These facilities commonly consist of holders (bamboo and wood structure or galvanized steel pipe structure) and a covering membrane (made of polyethylene or another material). Grape rain-shelter cultivation developed from short pruning and arched shed cultivation of Campbell Early in western Japan and was widely applied in Japan in the 1970s [3]. The technology came to China in the 1980s and has been increasing in the 1990s [4]. Currently, it is applied mainly to table grapes in the southern regions of China, which effectively overcomes rain problems, decreases the severity of diseases, improves fruit quality, and achieves satisfactory economic and social benefits [5]. Detoni *et al*. [6] found that Cabernet Sauvignon fruits under covering presented higher contents of titrable acidity and lower content of total anthocyanins than those picked from plants without the plastic covering in the South of Brazil; in the plants under shelter, the production was higher than that in the plants grown under open field conditions. Meanwhile, the plastic covering provided a decrease in the incidence of diseases. In China, similar results were obtained in the study from Wang *et al*. [7]. This suggests that the plastic covering makes possible the cultivation of “Cabernet Sauvignon” grapes in an organic production system.

Phenolic compounds are one of the major quality factors in wine grapes and in the resulting wine. They direct influence some important organoleptic characteristics of wines, such as color, flavor, bitterness and astringency [8]. Although phenolic compounds found in wine can also originate from microbial and oak sources, the majority of the phenolic compounds are grape-derived [9]. In grape berries, phenolic compounds are present mainly in skins and seeds. These compounds are extracted from grape skins and seeds into wines by maceration. Many factors may influence the phenolic composition of wines, such as grape variety, edaphoclimatic conditions, and cultural and technological practices [10,11,12,13,14,15]. As a new cultural and technological practice, previous studies have confirmed that rain-shelter cultivation can improve the grape quality and reduce the incidence of diseases in rainy regions [6,7]. However, to our knowledge, to date there is no published literature on the grape diseases and phenolic profiles in the skins of Cabernet Gernischet grape (*Vitis vinifera* L.) cultured under a rain-shelter model.

In view of the foregoing, the objective of the present study was to compare the phenolic profiles and incidence of diseases of Cabernet Gernischet grape cultured under rain-shelter and open-field conditions. From a practical standpoint, this study was carried out to provide sufficient experimental evidence for further application and diffusion of rain-shelter cultivation.

## 2. Results and Discussion

### 2.1. Effect of Rain Shelter on Incidence of Grape Diseases

Rain-shelter cultivation has been applied widely in some rainy areas, such as China, Japan, Brazil, *et al.*, where diseases become serious during the fruit ripening period due to high rainfall and air humidity. Excessive rainfall is responsible for its harmful effects. Prolonged wet weather through ripening increases the risk from diseases such as downy mildew and botrytis rots [16]. Direct rain damage to ripening berries results from their rapid swelling due to water intake, which is related to the osmotic pressure developed in the juice with rising sugar content [17]. It can come from the roots and directly through the leaves or berry skins. Cracking, compression and sometimes ejection from the bunch occurs and then is readily followed by botrytis and other rotting. It is worst when previous conditions have been dry, because the berry skins are then more brittle and relative swelling may be greater [16]. The direct contact between grapevines and rainwater can be effectively avoided using rain-shelter cultivation. Table 1 and Table 2 show the diseases of grape leaves and fruits under rain-shelter cultivation and open-field cultivation during ripening for the 2010 and 2011 vintages, respectively. In this study, there no pesticides were sprayed during the experimental process to keep consistent experimental conditions. Therefore, these grapevines cultured on the open field presented serious diseases and the severity increased to the forth grade (infected area of leaves is above 75%). The diseases of infected plants were found mainly to involve downy mildew on grape leaves, anthracnose and white rot in grape berries. These diseases could proliferate and spread during the hot and rainy season. In the two vintages, leaf disease (disease incidence of leaf, defoliation rates, and diseases index) and fruit disease (diseases incidence of cluster, diseases incidence of berry, and diseases index) in these grapevines cultured using rain-shelter technology were far lower than those cultured on the open field during the fruit ripening process. These results are in agreement with those described previously by Detoni *et al*. [6], who found that high incidence of diseases in plants without plastic covering caused lower production compared to those covered by plastic. Thus, application of rain-shelter cultivation could indirectly reduce the dependence on chemical pesticides in the grape industry, which makes possible the cultivation of grape “Cabernet Gernischet” in an organic production system, by providing a decrease in the incidence of diseases.

### 2.2. Effect of Rain Shelter on Grape Berry Maturity

**Figure 1:**
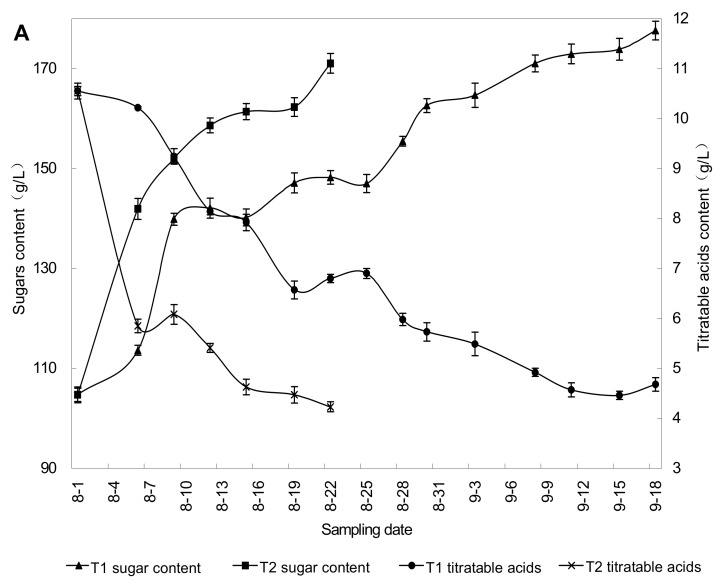

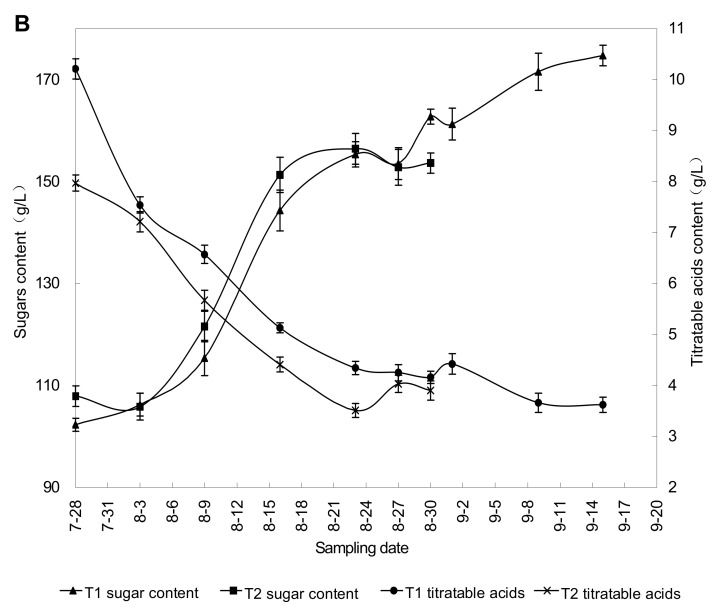
Changes of sugar and titratable acids in grape berry during ripening period in the 2010 (**A**) and 2011 (**B**) vintage.

The microclimate factors of vineyards under rain-shelter cultivation such as air temperature and humidity, photosynthetic radiation, wind speed, and soil moisture content was altered compared to those under open-field cultivation [18,19,20]. It has been confirmed that the impermeable plastic covering above the grapevines rows increased the air temperature and decreased the photosynthetic radiation and wind speed. The covering interfered with the quality of the incoming solar radiation, mainly by reducing the irradiance in the ultraviolet band and also by reducing the ratio between the irradiance in the red and far-red bands [18,19]. Table 3 shows the microclimatic parameters in the canopy of Cabernet Gernischet grape under rain-shelter and open-field cultivation in the present study. Similar to previous studies [18,19,20], the temperature and relative humidity in the canopy of Cabernet Gernischet grape under rain-shelter cultivation were elevated compared to open-field cultivation, while the illumination intensity was declined. The climate conditions, especially sunlight and temperature, have significant effects on the berry growth and development [16]. Figure 1 showed that the accumulation rate of sugar and degradation rate of titratable acids in the grapes under rain-shelter cultivation declined obviously compared to open-field cultivation, leading the fruit maturation to postpone. That is consistent with the studies of Detoni *et al.* [6] and Chavarria *et al.* [21]. It could be due to the changed microclimate of vineyards under rain-shelter cultivation. The results from Bergqvist *et al.* [22] suggest that the effects of light on fruit composition are heavily dependent upon the extent to which berry temperature is elevated as a result of increased sunlight exposure. Sugar and pigments can peak four to five weeks from veraison in a warm and sunny environment, after which flavor ripening starts and lasts for three weeks. However in more cloudy climate, flavor ripening and sugar accumulation can be roughly synchronous, while in still more cloudy climates such as in Northern Europe the build-up of sugar can lag, leading to flavor maturity at low sugar levels [16].

### 2.3. Effect of Rain Shelter on Phenolic Profile of Berry Skins

Phenolic compounds, an important group of plant secondary metabolites, are present in large amounts in fruits of different *Vitis* species, among which *V. vinifera* is the most representative. These compounds are also abundant in one of the end products of grape, *i.e.*, wine. They play an important role in the quality of grapes and wines [23,24,25]. These constituents can be divided into two groups: flavonoid compounds (anthocyanins, flavan-3-ols and flavonols) and non-flavonoid (hydroxybenzoic and hydroxycinnamic acids and stilbenes). The content of phenolic compounds in grape berries depends on climatic and geographical factors, cultural practices, and grape cultivar. For a better characterization of the phenolic composition of Cabernet Gernischet grape under rain-shelter cultivation, a detailed study by HPLC-MS/MS was performed. Finally, thirty-six phenolic compounds were identified, including 13 anthocyanins, 11 flavonols, six flavan-3-ols, two stilbenes, three hydroxybenzoic acids and one hydroxycinnamic acid (Table 4).

Anthocyanins are mainly responsible for color in grapes and young wines [26,27]. They mostly exist in grape skins and are transferred to wine through maceration during alcoholic fermentation [9]. Malvidin monoglucoside and its derivatives were the dominant anthocyanins in the two vintages of grape berries and accounted for an average of 82–90% of the total anthocyanin compounds identified, which is consistent with Cabernet Gernischet grape from the foot of Qilian Mountain on the ancient Silk Road in northwest China [28]. Unlike previous study on Cabernet Gernischet grape [28], delphinidin-3-*O*-(6-*O*-coumaroyl)-glucoside, cyanidin-3-*O*-(6-*O*-acetyl)-glucoside and cyanidin-3-*O*-(6-*O*-coumaroyl)-glucoside were not identified in the four samples from two vintages in present study, which may be connected to local climatic and geographical factors. For most anthocyanins, rain-shelter cultivation reduced their content in the grape skins as a whole. Accordingly, the total anthocyanin content in the grape under rain-shelter cultivation were also lower compared to open-field, which was consistent with previous studies [6,7].

**Table 4:** Phenolic profiles of berry skins under rain-shelter cultivation (T1) and open-field cultivation (T2) in the 2010 and 2011 vintages.

Phenolic compound	[M^+^]/[M-H]^−^ (Frag. MS^2^ *m/z*)	Vintage 2010		Vintage 2011	
		T1	T2	T1	T2
**Anthocyanins (mg ME/kg DW)**					
Delphinidin-3-*O*-glucoside	465 (303)	686.03 ± 50.84 ^b^	1055.62 ± 85.37 ^a^	462.09 ± 40.27 ^b^	623.96 ± 59.64 ^a^
Delphinidin-3-*O*-(6-*O*-acetyl)-glucoside	507 (303)	236.87 ± 20.67 ^b^	373.17 ± 15.84 ^a^	nd	152.18 ± 17.96
Petunidin-3-*O*-glucoside	479 (317)	682.10 ± 73.49 ^b^	1126.87 ± 60.35 ^a^	523.99 ± 40.54 ^b^	664.54 ± 52.67 ^a^
Petunidin-3-*O*-(6-*O*-acetyl)-glucoside	521 (317)	173.63 ± 21.44 ^b^	327.61 ± 33.72 ^a^	106.56 ± 17.33 ^b^	246.49 ± 20.67 ^a^
Petunidin-3-*O*-(6-*O*-coumaroyl)-glucoside	625 (317)	336.26 ± 16.56 ^a^	52.27 ± 3.78 ^b^	nd	nd
Cyanidin-3-*O*-glucoside	449 (287)	90.70 ± 7.45 ^a^	72.47 ± 8.30 ^b^	41.72 ± 5.34 ^a^	41.93 ± 3.33 ^a^
Peonidin-3-*O*-glucoside	463 (301)	462.35 ± 12.84 ^a^	455.16 ± 20.35 ^a^	318.21 ± 25.84 ^a^	295.78 ± 16.47 ^ab^
Peonidin-3-*O*-(6-*O*-acetyl)-glucoside	505 (301)	406.28 ± 51.35 ^b^	703.86 ± 47.84 ^a^	211.29 ± 14.67 ^a^	180.44 ± 15.45 ^b^
Peonidin-3-*O*-(6-*O*-coumaroyl)-glucoside	609 (301)	50.14 ± 6.84	nd	nd	nd
Malvidin-3-*O*-glucoside	493 (331)	5964.82 ± 231.42 ^b^	8975.48 ± 476.53 ^a^	6206.61 ± 366.67 ^b^	7444.34 ± 425.56 ^a^
Malvidin-3-*O*-(6-*O*-acetyl)-glucoside	535 (331)	2937.81 ± 118.48 ^b^	5380.51 ± 234.84 ^a^	3174.80 ± 102.43 ^b^	3729.39 ± 229.33 ^a^
Malvidin-3-*O*-(6-*O*-coumaroyl)-glucoside	639 (331)	5340.91 ± 346.76 ^b^	9299.04 ± 513.90 ^a^	4677.49 ± 194.33 ^a^	3800.57 ± 260.65 ^b^
Malvidin-3-*O*-(6-*O*-caffeoyl)-glucoside	655 (331)	nd	673.65 ± 41.14	308.05 ± 37.24 ^b^	395.37 ± 29.56 ^a^
Sum of anthocyanins		17385.36 ± 1556.34 ^b^	28524.33 ± 3011.30 ^a^	16167.67 ± 837.67 ^b^	17575.00 ± 964.35 ^a^
**Flavan-3-ols (mg CE/kg DW)**					
(+)-Catechin	289 (245, 205, 179)	59.40 ± 4.89 ^b^	86.17 ± 10.84 ^a^	61.63 ± 6.31 ^a^	68.72 ± 4.54 ^a^
(-)-Epicatechin	289 (245, 205, 179)	32.87 ± 3.92 ^b^	59.23 ± 4.76 ^a^	33.77 ± 2.67 ^b^	69.37 ± 5.23 ^a^
Gallocatechin	305 (179)	61.32 ± 3.87 ^a^	35.39 ± 4.12 ^b^	15.61 ± 1.34 ^b^	17.41 ± 0.84 ^a^
dimer(epi)gallocatechin-(epi)catechin	593 (425)	0.53 ± 0.08 ^b^	0.75 ± 0.10^a^	tr	0.50 ± 0.10
Procyanidin dimmer	577 (425, 407, 289)	217.77 ± 16.45 ^a^	225.56 ± 10.89 ^a^	117.62 ± 12.89 ^a^	125.35 ± 14.36 ^a^
Procyanidin trimer	865 (713, 577, 289)	209.83 ± 12.35 ^b^	241.15 ± 16.45 ^a^	nd	142.09 ± 10.69
Sum of flavan-3-ols		581.72 ± 30.67 ^b^	648.23 ± 40.56 ^a^	228.61 ± 15.82 ^b^	423.44 ± 24.45 ^a^
**Flavonols (mg QE/kg DW)**					
isorhamnetin-3-*O*-glucoside	477 (315)	506.931 ± 36.48 ^a^	533.86 ± 41.22 ^a^	nd	nd
isorhamnetin-3-*O*-galactoside	477 (315)	12.23 ± 1.45^b^	25.72 ± 1.76^a^	nd	tr
**Flavonols (mg QE/kg DW)**					
quercetin-3-*O*-hexoside	463 (301)	nd	13.45 ± 0.97	32.82 ± 2.34	nd
quercetin-3-*O*-glucuronide	477 (301)	147.67 ± 13.33 ^a^	136.18 ± 14.52 ^a^	86.05 ± 6.67 ^a^	119.15 ± 10.48 ^a^
quercetin-3-*O*-galactoside	463 (301)	333.57 ± 29.35 ^a^	319.57 ± 20.67 ^a^	204.88 ± 19.59 ^b^	329.10 ± 18.84 ^a^
quercetin-3-*O*-rutinoside	609 (301)	254.88 ± 13.33 ^b^	286.73 ± 19.56 ^a^	91.99 ± 11.48 ^b^	166.04 ± 14.57^a^
dihydroquercetin-3′-*O*-rhamnoside	449 (303)	207.60 ± 15.27 ^a^	125.78 ± 10.44 ^b^	84.63 ± 6.42 ^a^	60.49 ± 10.76 ^b^
laricitrin-3-*O*-galactoside	493 (331)	92.11 ± 16.46 ^a^	47.99 ± 6.79 ^b^	23.27 ± 3.46 ^a^	23.90 ± 2.32 ^a^
laricitrin-3-*O*-glucoside	493 (331)	164.39 ± 28.54 ^b^	241.15 ± 30.21 ^a^	72.54 ± 6.48 ^b^	100.59 ± 14.39 ^a^
myricetin-3-*O*-galactoside	479 (317)	253.73 ± 11.25 ^b^	286.31 ± 13.47 ^a^	238.61 ± 20.36 ^a^	224.28 ± 17.54 ^a^
kaempferol-3-*O*-galactoside	447 (285)	76.22 ± 7.26 ^b^	114.28 ± 10.44 ^a^	197.26 ± 26.48 ^b^	337.65 ± 36.48 ^a^
Sum of flavonols		2049.31 ± 94.48 ^b^	2131.01 ± 106.25 ^a^	1032.04 ± 84.29 ^b^	1361.19 ± 116.84 ^a^
**Stilbenes (mg RE/kg DW)**					
*trans*-resveratrol	227 (185, 159)	7.72 ± 0.76 ^b^	11.63 ± 1.27 ^a^	4.36 ± 0.48 ^b^	8.69 ± 1.45 ^a^
*trans*-Piceid	389 (227)	5.64 ± 1.49 ^a^	7.12 ± 1.74 ^a^	4.21 ± 0.85 ^b^	7.96 ± 1.44 ^a^
Sum of stilbenes		13.36 ± 1.54 ^b^	18.75 ± 1.63 ^a^	8.57 ± 1.01 ^b^	16.65 ± 2.46 ^a^
**Hydroxybenzoic acids (mg GAE/kg DW)**					
hexose ester of vanillic acid	329 (167)	54.66 ± 8.48 ^b^	80.86 ± 9.38 ^a^	31.05 ± 1.56 ^a^	31.94 ± 3.00 ^a^
Syringic acid	197 (153)	91.83 ± 11.23 ^a^	103.24 ± 16.19 ^a^	105.40 ± 20.58 ^a^	126.07 ± 29.34 ^a^
hexose ester of protocatechuic acid	315 (153)	4.79 ± 0.52 ^a^	3.55 ± 0.38 ^a^	0.28 ± 0.05	nd
Sum of hydroxybenzoic acids		150.02 ± 10.38 ^b^	188.87 ± 21.67 ^a^	136.74 ± 18.49 ^a^	158.01 ± 20.21 ^a^
**Hydroxycinnamic acids (mg CAE/kg DW)**					
hexose ester of ferulic acid	355 (193)	2.16 ± 0.28 ^b^	4.41 ± 0.33 ^a^	3.13 ± 0.97 ^a^	4.08 ± 0.89 ^a^
Sum of hydroxycinnamic acids		2.16 ± 0.28 ^b^	4.41 ± 0.33 ^a^	3.13 ± 0.97 ^a^	4.08 ± 0.89 ^a^
Sum of flavonoids		20016.39 ± 1996.38 ^ab^	31303.57 ± 2910.86 ^a^	17428.32 ± 1489.65 ^b^	19359.63 ± 1480.46 ^a^
Sum of nonflavonoids		165.53 ± 17.43 ^b^	212.02 ± 10.67 ^a^	148.45 ± 20.37 ^ab^	178.74 ± 16.33 ^a^
Sum of phenolics		20181.92 ±2053.76 ^ab^	31515.59 ± 3219.34 ^a^	17576.77 ± 1524.63 ^b^	19538..37 ± 1805.33 ^a^

Values are means of duplicate determination ± S.D. nd, means not detected. tr, means trace. Different letters in each row of total concentrations are significantly different at the 0.05 level according to ANOVA.

Previous study showed that sunlight and temperature could promote the accumulation of anthocyanins in the grape berry [22]. Meanwhile high air humidity is detrimental to the anthocyanin accumulation [16]. Although increasing diurnal air temperatures, the plastic covering can reduce solar radiation and the wind velocity, and increase the air humidity around the grape berry by reducing the evaporative demand on vineyards [18,19]. It suggested that solar radiation and air humidity had higher influence on the anthocyanin accumulation than air temperatures during grape berries maturation.

Flavan-3-ols mainly contribute to the bitterness, astringency, and wine structure, and they play vital roles in the stabilization of wine color during aging [29]. They are found in the solid parts of the berry (seed, skin, and stem) in the form of monomers, oligomers, or polymers and move to musts and wines during winemaking [9]. In all of the grape samples both under rain-shelter cultivation and open-field cultivation, flavan-3-ols oligomer (procyanidin dimer and trimer) content was higher than monomer. Compared to grape berries under open-field cultivation, these grape berries under rain-shelter cultivation had low concentrations of proanthocyanidins. This may be mainly related to growth environmental conditions. Previous studies have confirmed that higher sun exposure positively influenced proanthocyanidin concentration [30,31]. Low sun exposure under rain shelter may cause the proanthocyanidins to drop during grape berries maturation. In grapes, the biosynthesis of flavanol monomers involves leucoanthocyanidin reductase (LAR) and anthocyanidin reductase (ANR), responsible for the synthesis of (+)-catechin and (−)-epicatechin, respectively. The expression of these enzymes is mainly related to grape cultivar and environmental conditions of vineyard [32]. Considering that the ratio (+)-catechin/(−)-epicatechin varies between the grapes under rain-shelter cultivation and open-field cultivation, it could be possible to confirm the variation in the LAR and ANR activity due to the effect of the microclimate around the grape clusters. Independent from the zone, it is possible to suppose that the LAR enzyme is more active than the ANR in the grapes under rain-shelter cultivation. In addition, the grapes cultivated under rain shelter had lower sum of flavan-3-ols content, which may be caused by the same reason with total anthocyanins. However, sum of flavan-3-ols content in present study were notably above that in Cabernet Gernischet grape from the foot of Qilian Mountain on the ancient Silk Road in northwest China [28].

**Figure 2:**
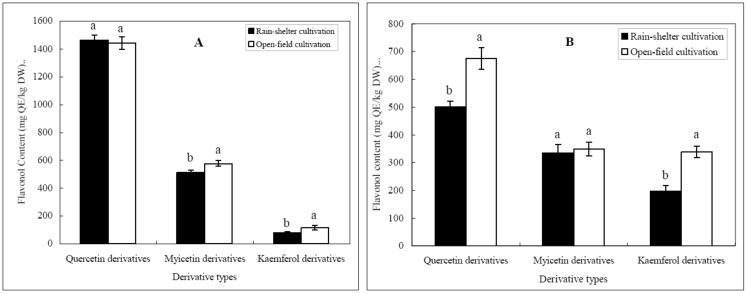
Flavonols distribution of grape samples in 2010 (**A**) and 2011 (**B**) vintage.

The last group of flavonoids is the flavonols, which seem to be responsible for bitterness and color [11]. They also contribute to the color stabilization of red wines by reinforcing the pigmentation due to anthocyanins, a phenomenon known as co-pigmentation [33]. Generally, they originate from the berry skins of grapes, and are transferred to the wine during the process of winemaking. In present study, rain-shelter cultivation lowered the level of total flavonols in grape berries, which may be caused by low solar radiation and high air humidity [18,19]. Based on the differences in their biosynthesis pathway in grape berries, flavonols could be classified into three groups: kaemferol derivatives, quercetin derivatives and myicetin derivatives. Their content in grape berries is shown in Figure 2. It was easily found that flavonol profiles were dominated by quercetin derivatives. It suggests the directional flow of carbon to the quercetin synthetic branch. In addition, dihydroquercetin-3-rhamnoside was detected in all the grape samples and its content in grape samples under rain-shelter cultivation was significantly higher than those under open-field cultivation. Considering that dihydroflavonols (flavanonols) are precursors of flavonols [32], the high concentration of dihydroflavonols and low concentration of flavonol could be related with a lower activity for flavonol synthase (FLS) of grapes under rain-shelter cultivation compared with those under open-field cultivation. With regard to the sum of flavonols, their content in present study rang from 1032.04 to 2131.01 mg QE/kg DW, which was significantly higher than that in Cabernet Gernischet grape from the foot of Qilian Mountain on the ancient Silk Road in northwest China [28], and Syrah grape from the Maipo Valley of Chile.

The non-flavonoids in grape and wine mainly include stilbenes and phenolic acids. Stilbenes are the important phenolic compounds because of their putative protective effects against cardiovascular diseases [34]. The concentrations of these compounds in wines depend on multiple factors such as grape variety, fungal infections, winemaking procedures, and weather conditions [35]. In this study, only *trans*-resveratrol and its 3-glucoside (*trans*-piceid) were identified and their contents are similar. Compared to the grape samples under open-field cultivation, the grape samples under rain-shelter cultivation had lower content of stilbenes. In grape and wine, there are two groups of phenolic acids; hydroxybenzoic acids and hydroxycinnamic acids. In this study, three hydroxybenzoic acids and one hydroxycinnamic acid were identified and quantified. Syringic acid was the most abundant phenolic acid in the grape samples analyzed. Similar to anthocyanins, flavan-3-ols and flavonols, rain-shelter cultivation also reduce the content of phenolic acids. In summary, rain-shelter cultivation decrease most phenolics accumulation during grape berries maturation.

## 3. Experimental

### 3.1. Plant Materials

The two-year study (2010 and 2011) was conducted at the Grape Demonstration Base of the College of Enology, Northwest A&F University, Jingyang County of Shaanxi Province, China (34°40′56″ N, 108°38′53″ E). The mesoclimate of vineyard during the growing season (from April to September) of the two vintages were shown in Table 5. Rows of own-rooted “Cabernet Gernischet” (*Vitis vinifera* L.) vines (planted in 2006) were oriented north-south on flat terroir with sandy soil. Vines were spaced to a spaced distance of 1.8 × 2.7 m and pruned to two buds per spur. Vines were trained to a bilateral cordon at 0.8 m above ground, in which shoots were trained upwards and each vine carried *ca.* 20 grape clusters. The vertical shoot-positioned canopies were uniformly managed. All vines were divided into two groups. Group one was cultured with rain-shelter cultivation technology. Shelters were built along with vines rows before berries coloration, and were 2.2 meter high, 1.7 meter wide and covered with colorless and transparent polyethylene film. Group two was a control and cultured on open field (open-field cultivation). The sugar content and titratable acids in grape berry were recorded during ripening. Clusters were harvested at their physiological maturity from the vineyards. Two groups of 100 berries of each treatment were randomly selected from the collected clusters and weighed. These samples were stored at −80 °C for subsequent analysis for their phenolic composition.

### 3.2. Chemicals and Standards

The standards of all phenolic compounds, including (+)-catechin, quercetin, gallic aid, caffeic acid, *trans*-resveratrol, and malvidin-3-*O*-glucoside, were supplied by Sigma-Aldrich (St. Louis, MO, USA). The purities of all the six standards were >97%. Methanol, formic acid, acetonitrile, and glacial acetic acid (HPLC grade) were obtained from Fisher Co. (Fairlawn, NJ, USA). Ethyl acetate (AR) was from Tianjin Bodi Chemical Reagent Co. Ltd. (Tianjin, China). All other chemicals used were analytical grade.

### 3.3. Determination of Some Basic Physicochemical Parameters

The sugar content and titratable acids were quantified according to the National Standard of the People’s Republic of China [36].

### 3.4. Diseases Investigation and Microclimate Evaluation

Thirty random shoots growing consistently from different parts of vines were investigated for disease incidence, disease index and defoliation rates; moreover, 30 random clusters from different parts of vines were investigated for disease incidence of cluster and berry, and disease index [2]. Grape disease was identified by visual inspection and investigated on 06/08/2010, 23/08/2010, 15/09/2010, 09/08/2011, 23/08/2011, and 15/09/2011, respectively. It was found mainly to be downy mildew in grape leaves of infected plants. Foliar symptoms of grape downy mildew appear as yellow circular spots with an oily appearance (oilspots). Young oilspots on young leaves are surrounded by a brownish-yellow halo. This halo fades as the oilspot matures. The spots are yellow or red in grape leaves. Under favorable weather conditions, large numbers of oilspots may develop and coalesce to cover most of the leaf surface. After suitably warm, humid nights, a white downy fungal growth (sporangia) will appear on the underside of the leaves.

In grape berries of infected plants, it was found mainly to be anthracnose and white rot. On fruit infected by anthracnose, lesions may be sunken and appear more reddish-black in color. As the lesions enlarge, the center will become increasingly sunken and turn gray. Fruit also may crack as the lesions expand. Berries affected directly by white rot show brown rot lesions. The fruit becomes abnormally juicy, shrivels, and brownish pustules appear over the surface.

The disease incidence and disease index were calculated by using the following formula:

(1) $$\mathrm{Diseases}\;\mathrm{incidence}\;(\%)=\frac{\mathrm{Infected}\;\mathrm{leaves}\;(\mathrm{cluster},\;\mathrm{berries})\;\mathrm{number}}{\mathrm{Investigated}\;\mathrm{leaves}\;(\mathrm{cluster},\;\mathrm{berries})\;\mathrm{number}}\times 100\;$$

(2) $$\mathrm{Diseases}\;\mathrm{index}=\frac{\sum \left[\mathrm{disease}\;\mathrm{grade}\times \mathrm{infected}\;\mathrm{leaves}\;\left(\mathrm{clusters},\;\mathrm{berries}\right)\;\mathrm{number}\right]}{\mathrm{Investigated}\;\mathrm{leaves}\;\left(\mathrm{clusters},\;\mathrm{berries}\right)\;\mathrm{number}\times \mathrm{highest}\;\mathrm{disease}\;\mathrm{grade}}\times 100\;$$

In the meantime, several microclimatic parameters (including average temperature, relative humidity and illumination intensity) in the canopy of Cabernet Gernischet grape under rain-shelter and open-field cultivation were recorded, respectively.

### 3.5. Extraction of Phenolic Compounds

For non-anthocyanin phenolics (including flavan-3-ols, flavonols, hydroxybenzoic acids, hydroxycinnamic acids, and stilbenes), triplicate samples of the pulverized berries skins of each treatment (2.00 g, dry weight) were exhaustively extracted four times with 5 mL of distilled water and 45 mL of ethyl acetate in a orbital shaker (SHZ-88A, Taicang Experiment Equipment Factory, Jiangsu, China) for 30 min at 20 °C to avoid thermal degradation. Then, these organic phases were combined and evaporated to dryness in a rotary evaporator (SENCO-R series; Shanghai Shensheng Biotech Co. Ltd., Shanghai, China) at 35°C under vacuum. Subsequently, the dried residuals were re-dissolved in 5 mL of methanol (HPLC grade). This methanol solution was filtered through a 0.45-μm organic membrane and analyzed by high performance liquid chromatography (HPLC) coupled with diode array detector (DAD) and electrospray ionisation mass spectrometry (ESI-MS).

For anthocyanins, triplicate freeze-dried skins ground (0.50 g, dry weight) were weighed into 50 mL centrifuge tube with 10 mL solvent (methanol/water/acetic acid, 70:29:1, v/v/v) in an orbital shaker at 300 rpm for 100 min at 25 °C. After pouring out the supernatant, the precipitate was re-extracted with the same solvent (10 mL) three times. The supernatant were combined in a 50 mL tube, and centrifuged at 8,000 rpm for 5 min. Finally the supernatant was collected and filtered through a 0.45-μm organic membrane. Finally, the resulting filtrates were used for qualitative and quantitative analyses of HPLC-DAD/ESI-MS.

### 3.6. HPLC-DAD/ESI-MS Analysis of Phenolic Compounds

The chromatographic analyses of non-anthocyanins were performed using an Agilent 1200 series LC-MSD trap XCT (Agilent Corporation, Santa Clara, CA, USA) equipped with a G1322A Degasser, a G1312B Bin pump, a G1367C HiP-ALS autosampler, a G1316B TCC (thermostated column compartment), a G1314C VWD (variable wavelength detector) and a reversed phase column (ZORBAX SB-C18, 3 × 50 mm i.d., 1.8 μm). The mobile phase consisted of (A) 1% acetic acid in water solution, and (B) 1% acetic acid in acetonitrile solution. The elution profile had the following proportions (v/v) of solvent B: 0.00–5.00 min, 5–8%; 5.00–7.00 min, 8–12%; 7.00–12.00 min, 12–18%; 12.00–17.00 min, 18–22%; 17.00–19.00 min, 22–35%; 19.00–21.00 min, 35–100%; 21.00–25.00 min, 100%; 25.00–27.00 min, 100–5%;. The column was held at 25 °C and was flushed at a flow rate of 1.0 mL min^−^^1^. The injection volume was 2 µL and analyses were detected at 280 nm. MS conditions were as follows: Electrospray ionization (ESI) interface, negative ion model, 35 psi nebulizer pressure, 10 mL min^−^^1^ dry gas flow rate, 325 °C dry gas temperature, and scans between *m/z* 100 and 1000 [37].

The chromatographic analyses of anthocyanins were performed using an Agilent 1100 series LC-MSD trap VL (Agilent Corporation) equipped with a G1379A Degasser, a G1311A quaternary pump, a G1313A ALS autosampler, a G1315B photodiode array detector and a reversed phase column (Kromasil C18, 250 × 4.6 mm i.d., 5 μm). The mobile phase consisted of (A) 6% (v/v) acetonitrile containing 2% (v/v) formic acid, and (B) 54% (v/v) acetonitrile containing 2% (v/v) formic acid. The elution profile had the following proportions (v/v) of solvent B: 0.00–1.00 min, 10%; 1.00–18.00 min, 10–25%; 18.00–20.00 min, 25%; 20.00–30.00 min, 25–40%; 30.00–35.00 min, 40–70%; 35.00–40.00 min, 70–100%; 40.00–45.00 min, 100–10%. The column was held at 50 °C and was flushed at a flow rate of 1.0 mL min^−^^1^. The injection volume was 30 µL. Diode array detection was performed from 200 to 900 nm and quantification was carried out by peak area measurements at 525 nm. MS conditions were as follows: Electrospray ionization (ESI) interface, positive ion model, 35 psi, 10 mL min^−^^1^ dry gas flow rate, 325 °C dry gas temperature, and scans between *m/z* 100 and 1000 [37].

All phenolic compounds was identified by comparison of their order of elution and retention time with those of standards and the weight of molecular ion and the fragment ion compared with standards and references [38,39,40,41,42]. Quantitative determinations were made by using the external standard method with the commercial standards. The calibration curves were obtained by injection of standard solutions under the same conditions as for the samples analyzed, over the range of concentrations observed. The compounds for which no standards were available were quantified with the curves of quercetin (flavonols and dihydroflavonols), *trans*-resveratrol (stilbenes), gallic acid (hydroxybenzoic acids), caffeic acid (hydroxycinnamic acids), (+)-catechin (flavan-3-ols) and malvidin-3-*O*-glucoside (anthocyanins). Therefore, flavonols, flavan-3-ols, hydroxybenzoic acids, hydroxycinnamic acids and stilbenes were respectively expressed as quercetin equivalence (QE), (+)-catechin equivalence (CE), gallic acid equivalence (GAE), caffeic acid equivalence (CAE), *trans*-resveratrol equivalence (RE), and malvidin-3-*O*-glucoside equivalence (ME) per gram of dry weight (DW). All of the analyses were performed in duplicate.

### 3.7. Statistical Analysis

Data were reported as mean ± standard deviation (SD) values of triplicate experiments, and were analyzed using DPS 7.55. One-way analysis of variance (ANOVA) and Duncan’s multiple range tests were used to determine the significance of the difference among samples, with a significance level of 0.05.

## 4. Conclusions

Grape diseases and phenolic profiles in the berry skins of *Vitis vinifera* cv. Cabernet Gernischet grape under rain-shelter cultivation were investigated and compared with those under open-field cultivation. Our results indicate that rain-shelter cultivation reduced the incidence of grape diseases significantly and delayed the maturation of Cabernet Gernischet fruits. Meanwhile, rain-shelter cultivation promoted dihydroquercetin-3-*O*-rhamnoside accumulation in grape skins during grape maturation. However, for most phenolic compounds identified, their content in grape samples under rain-shelter cultivation was lower than those under open-field cultivation. These were closely related to micrometeorological alterations in vineyards by using plastic covering under rain-shelter cultivation. It suggests the rain-shelter cultivation makes possible the cultivation of grape “Cabernet Gernischet” in the organic production system, for low incidence of diseases could reduce the application of chemical pesticide in grape and wine industry.

## Figures and Tables

**Table 1:** Diseases of grape leaves and fruits under rain-shelter cultivation (T1) and open-field cultivation (T2) during ripening in the 2010 vintage.

Disease parameters	August 6		August 22		September 15	
	T1	T2	T1	T2	T1	T2
**Leaves**						
Diseases incidence of leaves (%)	11.03 ± 4.56 ^a^	8.13 ± 3.27 ^a^	33.98 ± 3.62 ^b^	64.36 ± 7.67 ^a^	34.19 ± 7.84 ^b^	87.93 ± 5.39 ^a^
Defoliation rates (%)	—	—	—	—	2.05 ± 0.57 ^b^	41.92 ± 6.81 ^a^
Diseases index	3.73 ± 0.58 ^a^	1.97 ± 0.34 ^b^	6.91 ± 0.84 ^b^	38.94 ± 2.38 ^a^	9.48 ± 2.84 ^b^	71.48 ± 6.88 ^a^
**Fruits**						
Diseases incidence of cluster (%)	23.33 ± 2.14 ^b^	43.33 ± 3.47 ^a^	58.33 ± 6.12 ^b^	85.00 ± 6.33 ^a^	66.66 ± 3.27 ^b^	90.27 ± 5.56 ^a^
Diseases incidence of berry (%)	2.07 ± 0.46 ^a^	1.93 ± 0.32 ^a^	9.50 ± 1.64 ^b^	49.43 ± 4.26 ^a^	10.50 ± 1.38 ^b^	67.98 ± 4.84 ^a^
Diseases index	5.48 ± 0.46 ^a^	7.62 ± 0.94 ^a^	18.81 ± 2.56 ^b^	61.67 ± 4.55 ^a^	22.14 ± 1.89 ^b^	84.43 ± 3.22 ^a^

Values are mean ± S.D. values of three replicates. Diffrent letters in each row in a same investigating date are significantly different at the 0.05 level according to ANOVA by Duncan’s test. —, represent not analyzed. Table 2 is consistent with Table 1.

**Table 2:** Diseases of grape leaves and fruits under rain-shelter cultivation (T1) and open-field cultivation (T2) during ripening in the 2011 vintage.

Disease parameters	August 9		August 23		September 15	
	T1	T2	T1	T2	T1	T2
**Leaves**						
Diseases incidence of leaves (%)	54.22 ± 3.29 ^b^	85.30 ± 7.49 ^a^	51.10 ± 6.38 ^b^	87.29 ± 4.56 ^a^	25.42 ± 4.56 ^b^	92.04 ± 6.39 ^a^
Defoliation rates (%)	1.35 ± 0.56 ^a^	2.68 ± 0.71 ^a^	4.39 ± 0.32 ^b^	15.60 ± 1.26 ^a^	8.12 ± 0.47 ^b^	25.95 ± 1.67 ^a^
Diseases index	8.13 ± 0.63 ^b^	13.55 ± 1.56 ^a^	7.97 ± 0.78 ^b^	19.74 ± 2.56 ^a^	4.41 ± 0.46 ^b^	25.82 ± 2.25 ^a^
**Fruits**						
Diseases incidence of cluster (%)	16.67 ± 1.22 ^b^	28.33 ± 3.24 ^a^	21.67 ± 2.18 ^b^	35.00 ± 3.46 ^a^	53.33 ± 6.25 ^b^	84.00 ± 5.21 ^a^
Diseases incidence of berry (%)	0.65 ± 0.10 ^b^	1.70 ± 0.21 ^a^	1.05 ± 0.12 ^b^	4.75 ± 0.64 ^a^	3.97 ± 0.43 ^b^	35.00 ± 4.29 ^a^
Diseases index	2.62 ± 0.27 ^b^	5.71 ± 0.67 ^a^	2.86 ± 0.22 ^b^	9.29 ± 1.38 ^a^	14.05 ± 1.22 ^b^	78.00 ± 6.27 ^a^

**Table 3:** Microclimatic parameters in the canopy of Cabernet Gernischet grape under rain-shelter (T) and open-field cultivation (C), respectively.

Microclimatic variables	Vintage 2010		Vintage 2011	
	T	C	T	C
Average temperature in the canopy (°C)	27.62	26.49	26.97	25.64
Relative humidity in the canopy (%)	70.12	68.26	69.45	68.00
Illumination intensity in the canopy (×10^5^ lx)	0.046	0.130	0.057	0.169

**Table 5:** **Table** **5.** Mesoclimate of vineyard during the growing season of the 2010 and 2011 vintage.

Parameter	April	May	June	July	August	September
**Vintage 2010**						
Average temperature (°C)	13.2	19.9	25.4	27.2	24.7	21.3
Rainfall (mm)	41.0	42.7	23.8	77.0	145.1	85.9
Sunshine hours	170.0	150.5	219.6	158.0	145.4	152.1
**Vintage 2011**						
Average temperature (°C)	16.5	19.4	25.5	26.5	24.1	18.3
Rainfall (mm)	15.2	83.5	25.7	50.3	87.2	321.1
Sunshine hours	224.6	197.5	200.5	183.1	189.1	94.7
